# The impact of seasonal variation on the composition of the volatile oil of *Polyalthia suberosa* (Roxb.) Thwaites leaves and evaluation of its acetylcholinesterase inhibitory activity

**DOI:** 10.1186/s12906-024-04443-z

**Published:** 2024-04-12

**Authors:** Orchid A. Mahmoud, Iriny M. Ayoub, Omayma A. Eldahshan, Abdel Nasser B. Singab

**Affiliations:** 1 Department of Pharmacognosy, Faculty of Pharmacy, Ain Shams University, 11566-Abbassia, Cairo, Egypt; 2 Center for Drug Discovery Research and Development, Faculty of Pharmacy, Ain Shams University, Cairo, 11566 Egypt

**Keywords:** Acetylcholinesterase, Alzheimer’s disease, Seasonal variation, *Polyalthia suberosa*, Volatile oil

## Abstract

**Background:**

*Polyalthia suberosa* (Roxb.) Thwaites (Annonaceae) is a medicinal plant that has been reported for its various pharmacological potentials, such as its anti-inflammatory, analgesic, antioxidant, and neuropharmacological activities. This study aimed to analyze the leaf essential oils of *P. suberosa* (PSLO) collected in different seasons, to evaluate the acetylcholinesterase inhibitory activity, and to corroborate the obtained results via *in-silico* molecular docking studies.

**Methods:**

The leaf essential oils of *P. suberosa* collected in different seasons were analyzed separately by GC/MS. The acetylcholinesterase inhibitory activity of the leaves oil was assessed via colorimetric assay. In-silico molecular docking studies were elucidated by virtual docking of the main compounds identified in *P. suberosa* leaf essential oil to the active sites in human acetylcholinesterase crystal structure.

**Results:**

A total of 125 compounds were identified where D-limonene (0.07 − 24.7%), *α*-copaene (2.25 − 15.49%), E-*β*-caryophyllene (5.17 − 14.42%), 24-noroleana-3,12-diene (12.92%), *β*-pinene (0.14 − 8.59%), and *α*-humulene (2.49–6.9%) were the most abundant components. Results showed a noteworthy influence of the collection season on the chemical composition and yield of the volatile oils. The tested oil adequately inhibited acetylcholinesterase enzyme with an IC_50_ value of 91.94 µg/mL. Additionally, in-silico molecular docking unveiled that palmitic acid, phytol, *p*-cymene, and caryophyllene oxide demonstrated the highest fitting scores within the active sites of human acetylcholinesterase enzyme.

**Conclusions:**

From these findings, it is concluded that *P. suberosa* leaf oil should be evaluated as a food supplement for enhancing memory.

**Supplementary Information:**

The online version contains supplementary material available at 10.1186/s12906-024-04443-z.

## Introduction

Alzheimer’s disease (AD) is one of the challenging disorders of our century and is the root cause of dementia. Throughout the world, about 40 million people suffer from dementia, and this number is supposed to double as much every 20 years until approximately 2050 [1]. AD is a progressive, multifactorial, and neurodegenerative disorder. The pathology of AD may be attributed to several factors such as extracellular deposition of Aβ peptide and the intracellular aggregation of tau protein. In addition, the significant decrease in the neurotransmitter acetylcholine (ACh) in the brain is associated with the advancement of AD. Therefore, the enhancement of the central cholinergic function by acetylcholinesterase inhibition is one of the powerful ways to treat AD [2, 3]. Rivastigmine and tacrine, common drugs against AD possessing acetylcholinesterase (AChE) inhibitory activity, are reported to exert numerous side effects such as liver toxicity, nausea, and diarrhea [2]. Nature has provided us with foods and phytoconstituents that are valuable to human health. Nowadays, natural products attained much interest and played essential roles in the treatment of many diseases [4, 5]. In this context, galantamine isolated from plant source has been used as an AChE inhibitor in several countries with mild side effects [6].

Genus *Polyalthia*, belonging to family Annonaceae, comprises more than 100 species widely distributed in tropical and subtropical regions, including South Asia, South East Asia, and Australia [7]. Traditionally, *P. longifolia* has been used to treat many ailments such as fever, headache, high blood pressure, diabetes, and infections [8]. Fruits of *P. suberosa* have been used as anti-diarrheal. The leaves have been used to treat colds and coughs, and the barks have been used as analgesic [9]. Reported biological activities include antioxidant, anti-inflammatory, anticancer, antibacterial, and antiviral [7, 10, 11].

*Polyalthia suberosa* (Roxb.) Thwaites is an evergreen shrub traditionally used as laxative, abortifacient, analgesic, and in treatment of numerous skin infections. It was reported that different extracts of *P. suberosa* exerted anti-inflammatory, analgesic, anti-bacterial, antioxidant, and neuropharmacological activities [9, 10]. Despite the biological importance of this plant, the isolated essential oil from the leaves wasn’t fully investigated, only the cytotoxic and antimicrobial activities have been studied [12].

The chemical composition of plants is known to be affected by various extrinsic factors including climate, phenological phases, altitude, and soil. Thus, the chemical composition of essential oils and biological activity, which is dependent on the composition, are susceptible to variations [13, 14].

The present study was designed to investigate the composition of the volatile oils isolated from *P. suberosa* fresh leaves (PSLO), cultivated in Egypt, by Gas Chromatography/Mass Spectrometry (GC/MS) and the effect of seasonal variation on the yield and composition of these volatiles was assessed. Furthermore, the acetylcholinesterase inhibitory activity was evaluated. In addition, molecular docking was implemented to evaluate the binding affinities between the major oil components and acetylcholinesterase enzyme. ADMET prediction was carried out to evaluate the pharmacokinetics, pharmacodynamics, and toxicity properties of the identified compounds. This can help understand the possible use of PSLO as acetylcholinesterase inhibitor for incorporating this oil in formulations to treat AD.

## Materials and methods

### Plant material

The fresh leaves of *P. suberosa* were collected from Zoo Garden, Giza, Egypt; and was identified and authenticated by Mrs Therese Labib, Plant Taxonomy Consultant at Ministry of Agriculture and El-Orman Botanical Garden, Giza, Egypt. Leaves were collected in four seasons (2019 and 2020); namely winter (January), spring (April), summer (August), and autumn (November). A voucher specimen of the plant (PHG-P-PS-297) was stored at the Pharmacognosy Department, Faculty of Pharmacy, Ain Shams University, Cairo, Egypt.

### Isolation of volatile constituents

Fresh leaves of *P. suberosa* (200 g), collected in four different seasons, were exposed to six hours of hydrodistillation using a Clevenger-type apparatus. The oil yields were measured in %*w/w* based on the initial weight of the plant. The oils were kept for further analysis in sealed vials at 4 °C.

### GC/MS analysis

GC/MS analysis was carried out using Shimadzu GC/MS-QP 2010 (Kyoto, Japan) supplied with Rtx-5MS capillary column (30 m x 0.25 mm i.d. x 0.25 *μ*m film thickness) (Restek, USA). The initial oven temperature was kept at 45 °C for 2 min (isothermal), then heated to 300 °C at a rate of 5 °C/min and kept constant at 300 °C for 5 min (isothermal). The injector temperature was held at 250 °C. The used carrier gas was helium using a constant flow rate of 1.41 mL/min. An auto sampler was used to inject the sample (1 µL) and the split ratio was 15:1. The MS conditions were as follows: (equipment current) filament emission current: 60 mA, ion source temperature: 200 °C, ionization voltage: 70 eV and scan range: 35–500 amu [15].

### Identification of volatile constituents

Identification of volatile constituents was achieved by comparison of their retention indices, their mass spectra and fragmentation patterns with the National Institute of Standards and Technology (NIST-17) database, Adams, and literature [16–25]. Retention indices (RI) were deduced relative to a homologous series of n-alkanes (C8-C28) injected under the same conditions as the essential oils [26].

### Acetylcholinesterase inhibitory activity

Acetylcholinesterase inhibitory activity was assessed according to the method of Ellman et al. [27] with some modifications. AChE was obtained from *Electrophorus electricus.* 3,3′-Dithiodipropionic acid di-(N-hydroxysuccinimide ester) (DTNB) was used as an indicator. The indicator solution (10 µL) was transferred to a 96-well plate, followed by 20 µL of the enzyme solution, then 20 µL of oil samples at a concentration range of (100-5 µg/mL) were added. Afterwards, 10 µL of the substrate acetylcholine iodide was added to all wells. The plate was incubated in a dark chamber for 20 min at room temperature. At the end of the incubation period, the color was measured at 412 nm using a microplate reader. Donepezil was used as a positive control. Percentage inhibition was calculated using this formula:

$$\begin{array}{l} \text{P}\text{e}\text{r}\text{c}\text{e}\text{n}\text{t}\text{a}\text{g}\text{e}\,\text{i}\text{n}\text{h}\text{i}\text{b}\text{i}\text{t}\text{i}\text{o}\text{n}=\\\frac{\left(\text{A}\text{b}\text{s}\text{o}\text{r}\text{b}\text{a}\text{n}\text{c}\text{e}\,\text{o}\text{f}\,\text{c}\text{o}\text{n}\text{t}\text{r}\text{o}\text{l}-\text{a}\text{b}\text{s}\text{o}\text{r}\text{b}\text{a}\text{n}\text{c}\text{e}\,\text{o}\text{f}\,\text{s}\text{a}\text{m}\text{p}\text{l}\text{e}\right)}{\text{A}\text{b}\text{s}\text{o}\text{r}\text{b}\text{a}\text{n}\text{c}\text{e}\,\text{o}\text{f}\,\text{c}\text{o}\text{n}\text{t}\text{r}\text{o}\text{l} }\times 100\end{array}$$

IC_50_ (concentration of sample used to inhibit 50% of acetylcholinesterase under the test conditions) was calculated as previously reported [27, 28].

### Statistical analysis

Data was presented as mean ± SD of three measurements. The IC_50_ values were calculated by Microsoft Excel 2010 (level of significance *p* < 0.05).

### *In-silico* molecular docking study

#### Molecular docking study

Cholinesterase inhibitory activity was farther elucidated by virtual docking of the main compounds identified in PSLO to the active sites in human acetylcholinesterase (PDB ID: 4EY7, 2.35 Å) crystal structure, using Discovery Studio 2.5 (Accelrys Inc., San Diego, CA, USA). The complex of the human acetylcholinesterase crystal structure with donepezil, a pharmacologically active Alzheimer’s disease drug, was retrieved from the Protein Data Bank (http://www.rcsb.org/pdb/), accessed on 23rd October 2023. Donepezil, the co-crystallized inhibitor, was employed to identify the active binding sites in the AChE enzyme. The ligand was taken out before the docking simulations. Discovery Studio 4.5 (Accelrys Inc., San Diego, CA, USA) was utilized acquiring the C-docker protocol as previously described [15, 29–32]. To verify C-Docker as a docking algorithm, donepezil was removed from 4EY7, after that it was superimposed on the docked pose of the co-crystallized inhibitor and finally the root mean square deviation (RMSD) was calculated.

#### ADMET predictions

Absorption, distribution, metabolism, excretion, and toxicity (ADMET) were predicted for the main compounds identified in PSLO implementing ADMET prediction protocol in Discovery Studio 4.5 (Accelrys Inc., San Diego, CA, USA).

## Results and discussion

### GC/MS analysis

The essential oils of *P. suberosa* fresh leaves were prepared using hydrodistillation technique. All volatile oil samples were light yellow in color, displaying a characteristic odor. The oil yields of summer, autumn, winter, and spring were 0.03, 0.04, 0.02, and 0.07%*w/w*, respectively. The highest yield was obtained from leaves collected during spring (0.07%*w/w*) followed by summer (0.03%*w/w*) while the lowest yield was obtained during winter (0.02%*w/w*).

**Fig. 1:**
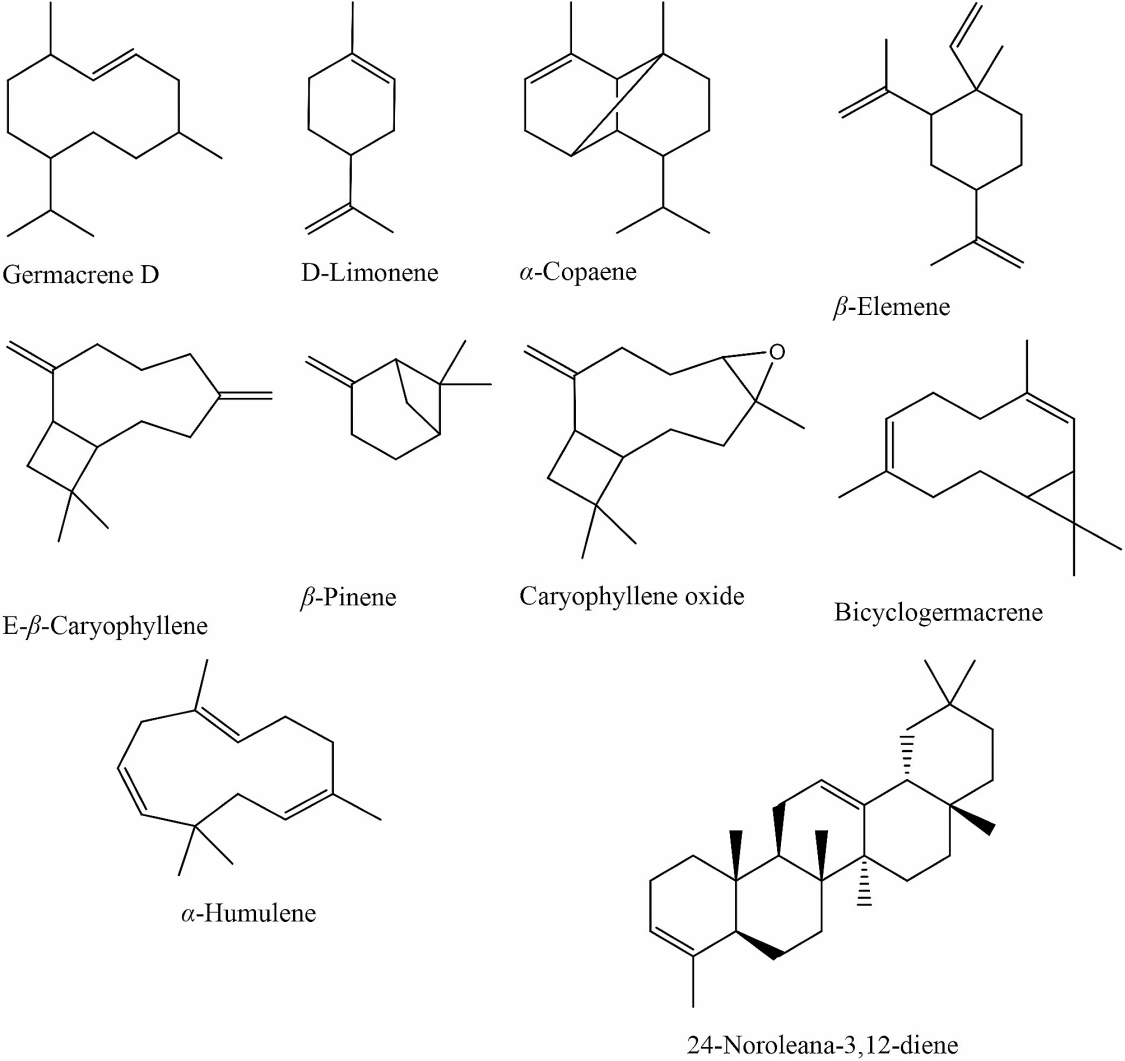
Structures of the major components identified in *P. suberosa* leaf volatile oil in different seasons

**Fig. 2:**
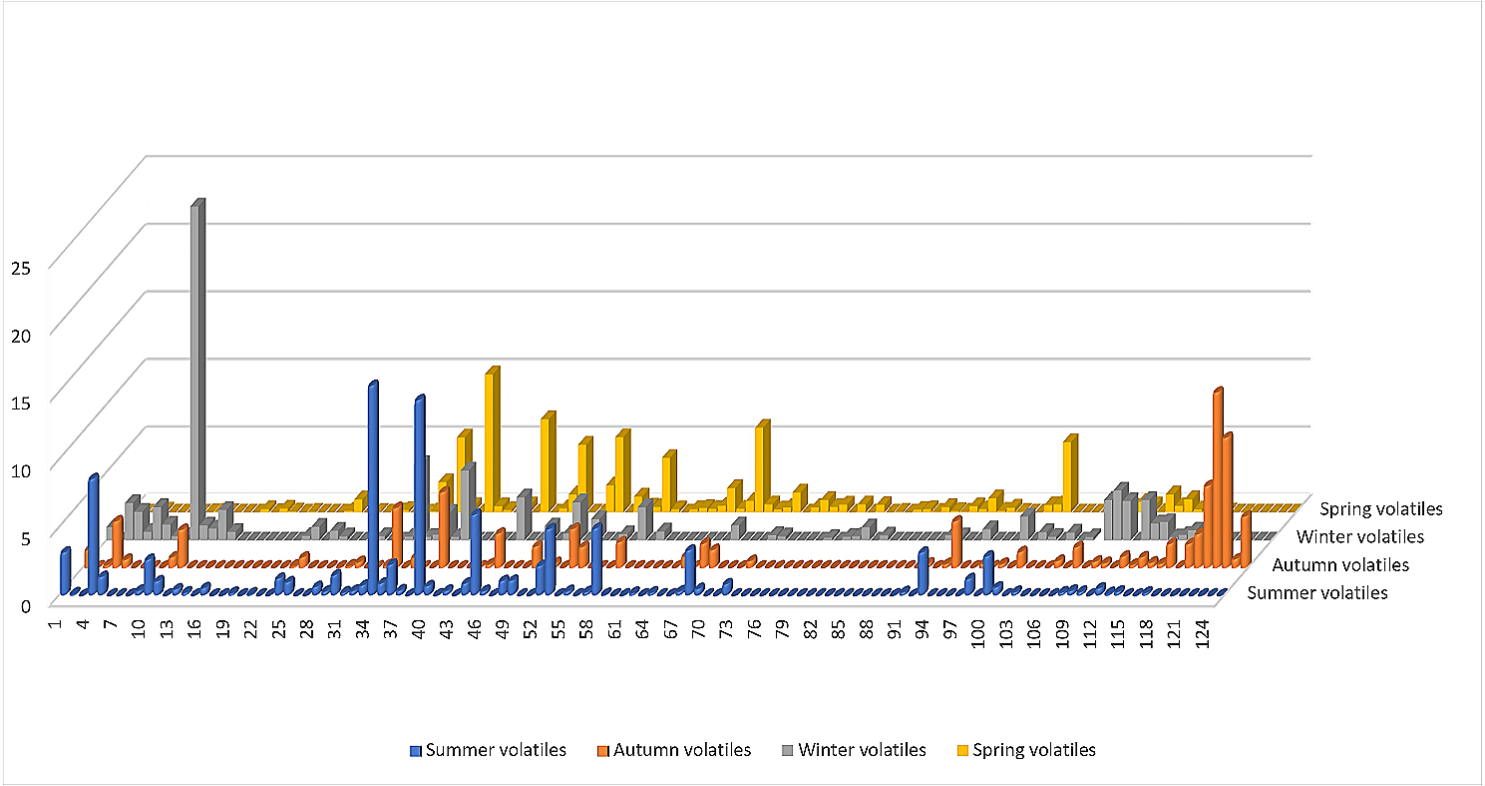
Bar chart of *P. suberosa* volatile constituents in four different seasons

GC/MS analyses of the volatile oil samples showed distinctive qualitative and quantitative differences. A meticulous comparative analysis of the oils is described in Table 1. The chemical structures of the major compounds are shown in Fig. 1. Identified compounds for the different samples accounted for 96.52, 85.5, 95.79, and 81.92% of the total composition in summer, autumn, winter, and spring seasons, respectively. A total of 125 compounds belonging to different classes were identified by comparing their mass fragmentation patterns and retention indices to the reported data and NIST library. A bar chart of the different components of *P. suberosa* volatile oils was constructed to show the qualitative and quantitative differences among the volatile samples (Fig. 2). GC/MS chromatograms of the leaves volatile oils of different seasons are shown in Fig. S1.

**Table 1:** Chemical profile of *Polyalthia suberosa* volatile oils in four different seasons

Peak no.	Rt	Compound	Molecular formula	RI _exp_	RI _lit_	Content (%)				Identification
						Summer	Autumn	Winter	Spring	
1	7.32	*α*-Pinene	C_10_H_16_	917	917	3.17	1.28	0.99	0.03	RI, MS
2	7.75	Camphene	C_10_H_16_	931	930	0.12	-	-	-	RI, MS
3	8.554	Sabinene	C_10_H_16_	961	961	-	0.16	2.77	0.03	RI, MS
4	8.65	***β*** **-Pinene**	C_10_H_16_	964	964	8.59	3.4	2.13	0.14	RI, MS
5	9.125	*β*-Myrcene	C_10_H_16_	981	981	1.36	0.56	0.65	-	RI, MS
6	9.67	*β*-cis-Ocimene	C_10_H_16_	1001	1002	-	-	2.47	-	RI, MS
7	9.88	2- Carene	C_10_H_16_	1007	1007	-	-	1.18	-	RI, MS
8	10.025	2,6-Dimethyl nonane	C_11_H_24_	1012	1022	-	-	0.32	-	RI, MS
9	10.15	*p*-Cymene	C_10_H_14_	1016	1016	0.37	-	0.35	0.02	RI, MS
10	10.266	**D-Limonene**	C_10_H_16_	1020	1020	2.63	0.74	24.7	0.07	RI, MS
11	10.9	*β*-Ocimene	C_10_H_16_	1040	1040	1.04	2.77	1.11	0.03	RI, MS
12	11.105	5-Methyl decane	C_11_H_24_	1047	1056	-	-	0.89	-	RI, MS
13	11.21	*γ*-Terpinene	C_10_H_16_	1050	1050	0.44	-	2.26	-	RI, MS
14	12.155	Terpinolene	C_10_H_16_	1081	1081	0.2	-	0.64	-	RI, MS
15	12.5	Linalool	C_10_H_18_O	1092	1092	-	-	-	0.25	RI, MS
16	12.595	Nonanal	C_9_H_18_O	1095	1094	0.53	-	-	0.03	RI, MS
17	14.9	Thujol	C_10_H_18_O	1169	1165	-	-	-	0.29	RI, MS
18	15.335	*α*-Terpineol	C_10_H_18_O	1183	1183	-	-	-	0.08	RI, MS
19	16.17	*β*-Cyclocitral	C_10_H_16_O	1211	1210	0.17	-	-	-	RI, MS
20	16.47	Citronellol	C_10_H_20_O	1221	1221	-	-	-	0.06	RI, MS
21	17.25	*β*-Cyclohomocitral	C_11_H_18_O	1248	1251	0.11	-	-	-	RI, MS
22	17.36	2,4-Dimethylundecane	C_13_H_28_	1252	1253	-	-	0.35	-	RI, MS
23	17.82	3-Methyldodecane	C_13_H_28_	1268	1270	-	0.11	1.02	-	RI, MS
24	18.045	Borneol acetate	C_12_H_20_O_2_	1277	1277	1.24	0.73	-	0.13	RI, MS
25	18.205	Dihydroedulan IIA	C_13_H_22_O	1282	1284	0.97	-	0.73	0.97	RI, MS
26	19.145	2,3,5,8-Tetramethyldecane	C_14_H_30_	1315	1318	-	-	0.31	-	RI, MS
27	19.215	E-Methyl geranate	C_11_H_18_O_2_	1317	1315	-	-	-	0.05	RI, MS
28	19.26	IsoDihydrocarveol acetate	C_12_H_20_O_2_	1319	1322	0.62	-	-	-	RI, MS
29	19.575	*δ*-Elemene	C_15_H_24_	1327	1327	0.3	0.11	-	0.09	RI, MS
30	19.92	*α*-Cubebene	C_15_H_24_	1342	1342	1.48	0.4	0.32	0.19	RI, MS
31	20.05	1,1,6-Trimethyl-1,2-dihydronaphthalene	C_13_H_16_	1345	1344	0.21	-	-	-	RI, MS
32	20.24	*cis*-Carvyl acetate	C_12_H_18_O_2_	1353	1352	0.35	-	-	-	RI, MS
33	20.4	Cyclosativene	C_15_H_24_	1359	1358	0.72	0.14	0.29	0.09	RI, MS
34	20.68	***α*** **-Copaene**	C_15_H_24_	1368	1368	15.49	4.38	5.66	2.25	RI, MS
35	20.86	(E)-*β*-Damascenone	C_13_H_18_O	1374	1380	0.87	-	0.27	-	RI, MS
36	21.1	***β*** **-Elemene**	C_15_H_24_	1384	1384	2.26	0.73	0.44	5.55	RI, MS
37	21.545	cis-*β*-Caryophyllene	C_15_H_24_	1398	1400	0.38	-	2.05	0.49	RI, MS
38	21.75	5,5-Diethylundecane	C_15_H_32_	1406	1408	-	-	0.26	-	RI, MS
39	21.925	**E-** ***β*** **-caryophyllene**	C_15_H_24_	1410	1411	14.42	5.55	5.17	10.21	RI, MS
40	22.095	*α*-Ionone	C_13_H_20_O	1420	1420	0.68	-	-	0.46	RI, MS
41	22.225	*γ*-Elemene	C_15_H_24_	1425	1425	-	-	-	0.19	RI, MS
42	22.34	Aromadendrene	C_15_H_24_	1429	1429	0.35	-	-	-	RI, MS
43	22.37	*α*-Guaiene	C_15_H_24_	1430	1433	-	-	-	0.53	RI, MS
44	22.635	Nerylacetone	C_13_H_22_O	1440	1445	0.87	0.22	-	-	RI, MS
45	22.815	***α*** **-Humulene**	C_15_H_24_	1445	1445	5.96	2.49	3.19	6.9	RI, MS
46	22.95	Neoclovene	C_15_H_24_	1453	1453	0.33	-	-	-	RI, MS
47	23.11	2,5-di-tert-Butyl-*p*-quinone	C_14_H_20_O_2_	1459	1466	-	-	-	0.31	RI, MS
48	23.295	*γ*-Gurjunene	C_15_H_24_	1467	1467	1.06	-	1.04	1.34	RI, MS
49	23.53	**Germacrene D**	C_15_H_24_	1473	1473	1.08	1.53	-	5.01	RI, MS
50	23.54	2-Methyl tetradecane	C_15_H_32_	1476	1467	-	-	0.53	-	RI, MS
51	23.6	3-Methyl tetradecane	C_15_H_32_	1479	1472		0.31	2.82	-	RI, MS
52	23.68	*β*-Selinene	C_15_H_24_	1482	1482	2.16	-	-	2.01	RI, MS
53	23.95	**Bicyclogermacrene**	C_15_H_24_	1492	1492	4.89	2.84	1.58	5.57	RI, MS
54	24.025	*α*-Farnesene	C_15_H_24_	1495	1496	-	1.5	-	-	RI, MS
55	24.085	*δ*-Guaiene	C_15_H_24_	1497	1500	0.32	-	-	1.18	RI, MS
56	24.28	2,4-di-*t*-Butylphenol	C_14_H_22_O	1505	1502	-	-	0.47	0.45	RI, MS
57	24.37	*γ*-Cadinene	C_15_H_24_	1509	1509	0.25	-	-	0.42	RI, MS
58	24.595	*δ*-Cadinene	C_15_H_24_	1514	1514	4.93	1.86	2.46	4.04	RI, MS
59	24.825	Cadine-1,4-diene	C_15_H_24_	1524	1524	0.07	-	-	0.24	RI, MS
60	24.81	2-Methyl pentadecane	C_16_H_34_	1526	1533	-	-	0.67	-	RI, MS
61	24.965	*α*-Cadinene	C_15_H_24_	1532	1533	-	-	-	0.25	RI, MS
62	25.12	*α*-Calacorene	C_15_H_20_	1535	1536	0.17	-	-	0.34	RI, MS
63	25.255	Elemol	C_15_H_26_O	1543	1543	-	-	-	0.27	RI, MS
64	25.375	Ledol	C_15_H_26_O	1548	1549	0.2	-	-	0.5	RI, MS
65	25.52	E-Nerolidol	C_15_H_26_O	1554	1554	-	0.8	-	1.81	RI, MS
66	25.87	1,3,7,11-Tridecatetraene, 4,8,12-trimethyl-, (3E,7E)	C_16_H_26_	1567	1573	-	-	-	0.28	RI, MS
67	26.05	Spathulenol	C_15_H_24_O	1570	1570	0.3	1.75		0.86	RI, MS
68	26.17	**Caryophyllene oxide**	C_15_H_24_O	1575	1575	3.32	1.28	1.12	6.27	RI, MS
69	36.375	Globulol	C_15_H_24_O	1584	1584	0.47	-	-	0.6	RI, MS
70	26.544	Humulene epoxide I	C_15_H_24_O	1594	1593	-	-	-	0.22	RI, MS
71	26.66	Virdiflorol	C_15_H_26_O	1598	1598	-	-	-	0.39	RI, MS
72	26.8	Humulene epoxide II	C_15_H_24_O	1604	1604	0.82	0.46	0.4	1.48	RI, MS
73	27.095	Hexadecane	C_16_H_34_	1617	1600	-	-	0.31	-	RI, MS
74	27.225	1-*epi*-Cubenol	C_15_H_26_O	1622	1625	-	-	-	0.39	RI, MS
75	27.553	*τ*-Cadinol	C_15_H_26_O	1637	1637	-	-	-	0.93	RI, MS
76	27.65	*δ*-Cadinol	C_15_H_26_O	1641	1641	-	-	-	0.42	RI, MS
77	28.118	1-Heptadecene	C_17_H_34_	1661	1673	-	-	-	0.59	RI, MS
78	28.14	Hexadecane, 2-methyl	C_17_H_36_	1662	1666	-	-	0.21	-	RI, MS
79	28.28	14-Hydroxy-9-*epi*-(E)-caryophyllene	C_15_H_24_O	1668	1668	-	-	-	0.6	RI, MS
80	28.545	3-Methyl hexadecane	C_17_H_36_	1680	1677	-	-	0.28	-	RI, MS
81	28.628	Heptadecane	C_17_H_36_	1684	1700	-	-	0.32	0.57	RI, MS
82	28.835	6,6-Diethyltetradecane	C_18_H_38_	1692	1695	-	-	0.98	-	RI, MS
83	29.055	1-Pentadecanal	C_15_H_30_O	1702	1702	-	-	-	0.06	RI, MS
84	29.82	7-Methyl heptadecane	C_18_H_38_	1735	1745	-	-	0.38	-	RI, MS
85	29.84	Mintsulfide	C_15_H_24_S	1736	1741	-	-	-	0.21	RI, MS
86	30.1	*α*-Cyperone	C_15_H_22_O	1748	1746	-	-	-	0.25	RI, MS
87	30.225	4-Methyl heptadecane	C_18_H_38_	1753	1749	-	-	-	0.06	RI, MS
88	30.858	Octadecane	C_18_H_38_	1780	1800	-	-	-	0.41	RI, MS
89	31.055	Phytane	C_20_H_42_	1789	1795	-	-	-	0.12	RI, MS
90	31.315	Hexadecanal	C_16_H_32_O	1800	1800	-	-	-	0.07	RI, MS
91	31.835	Hexahydrofarnesyl acetone	C_18_H_36_O	1828	1825	0.23	0.28	0.39	0.47	RI, MS
92	32.9	Nonadecane	C_19_H_40_	1880	-	-	-	0.31	0.25	MS only
93	33.59	Palmitic acid methyl ester	C_17_H_34_O_2_	1909	1910	3.16	0.25	-	1.05	RI, MS
94	34.37	Palmitic acid	C_16_H_32_O_2_	1947	1946	-	3.39	-	0.13	RI, MS
95	35.0	3-Methyl nonadecane	C_20_H_42_	1977	1974	-	-	0.84	0.36	RI, MS
96	35.5	Octadecanal	C_18_H_36_O	2001	2010	-	-	-	0.06	RI, MS
97	35.55	Verticilla-4(20),7,11-triene	C_20_H_32_	2004	2004	-	0.29	-	-	RI, MS
98	36.88	Methyl octadeca-9,12-dienoate	C_19_H_34_O_2_	2076	2075	1.2	-	-	-	RI, MS
99	36.93	3-Methyleicosane	C_21_H_44_	2079	2072	-	0.25	1.77	0.55	RI, MS
100	37.06	Oleic acid methyl ester	C_19_H_36_O_2_	2086	2086	2.88	-	-	0.6	RI, MS
101	37.335	*trans*-Phytol	C_20_H_40_O	2101	2103	0.59	1.13	0.62	5.2	RI, MS
102	37.435	Heneicosane	C_21_H_44_	2107	2100	-	-	0.28	-	RI, MS
103	37.46	Methyl stearate	C_19_H_38_O_2_	2108	2109	0.25	-	-	-	RI, MS
104	37.615	5-Ethyl-5-methylnonadecane	C_22_H_46_	2117	2111	-	-	0.59	-	RI, MS
105	37.81	Oleic acid	C_18_H_34_O_2_	2127	2120	-	0.47	-	-	RI, MS
106	38.22	5-Methyl heneicosane	C_22_H_46_	2150	2151	-	-	0.25	0.06	RI, MS
107	38.282	Isoincensole	C_20_H_34_O_2_	2153	2152	-	1.49	-	-	RI, MS
108	38.73	3-Methyl heneicosane	C_22_H_46_	2178	2175	0.21	-	3	0.44	RI, MS
109	40.5	3-Methyl docosane	C_23_H_48_	2276	2275	0.36	0.41	3.7	0.79	RI, MS
110	42.2	2-Methyl tricosane	C_24_H_50_	2369	2365	0.26	0.32	2.92	0.53	RI, MS
111	42.86	3,11-Dimethyl tricosane	C_25_H_52_	2405	2405	-	-	-	0.05	RI, MS
112	43.9	3-Methyl tetracosane	C_25_H_52_	2472	2473	0.51	0.8	2.99	1.34	RI, MS
113	45.49	3-Methyl pentaocsane	C_26_H_54_	2574	2573	0.14	0.19	1.26	0.49	RI, MS
114	47.015	3-Methyl hexacosane	C_27_H_56_	2672	2672	0.27	0.74	1.37	0.98	RI, MS
115	48.46	2-Methyl heptacosane	C_28_H_58_	2765	2761	-	0.36	0.24	0.22	RI, MS
116	49.0	Squalene	C_30_H_50_	2800	2808	-	0.27	0.42	0.14	RI, MS
117	49.875	2-Methyl octacosane	C_29_H_62_	2856	2858	0.19	1.73	0.75	0.48	RI, MS
118	51.255	7-Methyl nonacosane	C_30_H_62_	2944	2945	-	-	-	0.08	RI, MS
119	51.725	Campesterol	C_28_H_48_O	2974	-	-	1.72	-	-	MS only
120	51.99	24-Norursa-3,9(11),12-triene	C_29_H_44_	2991	-	-	2.45	-	-	MS only
121	52.185	24-Norursa-3,12-diene	C_29_H_46_	3004	-	-	6.01	-	-	MS only
122	52.83	**24-Noroleana-3,12-diene**	C_29_H_46_	3045	3057	-	12.92	-	-	RI, MS
123	52.97	Stigmasta-3,5-diene	C_29_H_48_	3054	-	-	9.58	-	-	MS only
124	56.39	*γ*-Sitosterol	C_29_H_50_O	3274	3290	-	0.6	-	-	RI, MS
125	56.82	24-Norursa-3,12-dien-11-one	C_29_H_44_O	3301	3351	-	3.75	-	-	MS only
**Monoterpene hydrocarbons**						18.79	9.13	39.25	0.32	
**Oxygenated Monoterpene**						2.49	0.73	-	0.86	
**Sesquiterpene hydrocarbons**						56.62	21.53	22.2	46.89	
**Oxygenated Sesquiterpene**						5.34	4.57	1.91	15.46	
**Diterpene hydrocarocarbons**						0.59	2.91	0.62	5.32	
**Non-terpenic compounds**						1.94	5.22	29.92	8.53	
**Non-terpenic oxygenated compounds**						8.02	4.11	-	2	
**Others**						2.73	37.3	1.89	2.54	
**Total % identified**						96.52	85.5	95.79	81.92	

The names of the components are in order of their elution from the Rtx-5MS column

Identification was based on comparison of the compounds’ mass spectral data (MS) and retention indices (RI) with those of NIST Mass Spectral Library (2017), Wiley Registry of Mass Spectral Data 8th edition and Adams

**Fig. 3:**
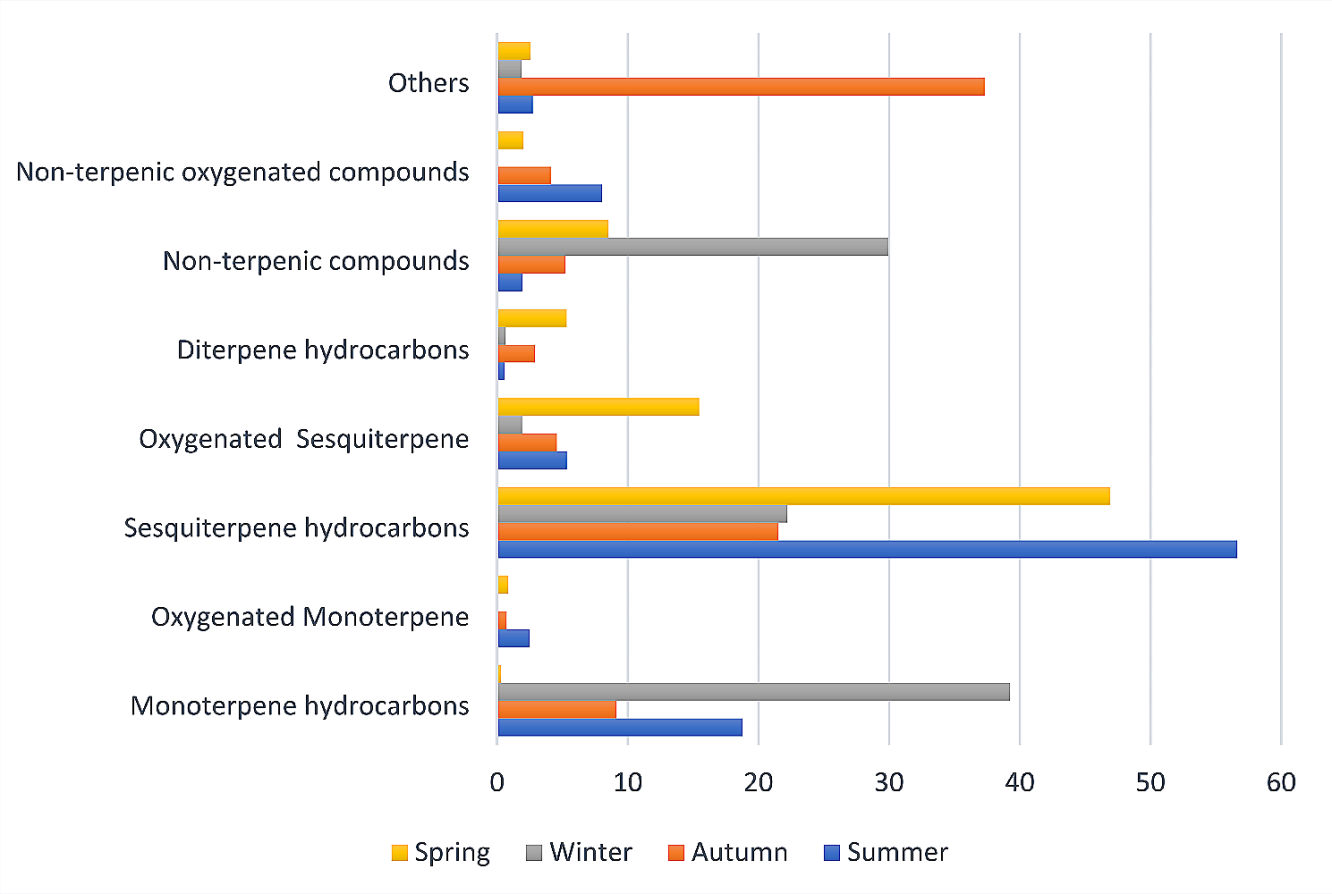
Seasonal variation of phytochemical classes observed in *P. suberosa* volatile oils

The identified compounds were classified into eight classes; as shown in Fig. 3 with sesquiterpene hydrocarbons being the most abundant class, ranging from 21.53 to 56.62% of the total volatile oils composition, where, the highest concentration was found in summer volatile oil (56.62%), followed by spring volatile oil (46.89%), then winter volatile oil (22.2%), and the lowest concentration was found in autumn volatile oil (21.53%). This finding was in accordance with previous reports, where sesquiterpene hydrocarbons (52.9–84.8%) were the predominant constituents of *P. sumatrana*, *P. stenopetalla*, *P. cauliflora*, and *P. rumphii* [33].

*α*-Copaene (2.25–15.49%), E-*β*-caryophyllene (5.17–14.42%), *α*-humulene (2.49–6.9%), bicyclogermacrene (1.58–5.57%), and *δ*-cadinene (1.86–4.93%) were the most abundant compounds of sesquiterpene hydrocarbons present in all seasons’ volatile oils. On the other hand, few compounds were detected only in a specific season. For example, *γ*-elemene (0.19%) and *α*-guaiene (0.53%) were detected in spring volatile oil only, meanwhile, aromadenderene (0.35%) was detected in summer volatile oil only. Our results were in accordance with the reported data in literature. For instance, *P. suberosa* leaf oil was reported to contain bicyclogermacrene (26.26%) and (E)-*β*-caryophyllene (7.79%) as the predominant constituents of the leaf oil [12]. The volatile oils of *P. harmandii, P. suaveolens*, and *P. longifolia var pendula* leaves contained major sesquiterpene hydrocarbons such as bicyclogermacrene (20.9%), *α*-humulene (34.2%), and E-*β*-caryophyllene (30%), respectively [34].

Monoterpene hydrocarbons were the second most abundant class of compounds, with the highest concentration detected in winter volatile oil sample (39.25%) and the least concentration detected in spring volatile oil (0.32%). D-limonene was the most abundant compound of this class with concentrations of 2.63, 0.74, 24.7 and 0.07% in summer, autumn, winter, and spring, respectively. *β*-Pinene was detected with the highest concentration in summer volatile oil (8.59%) and the least concentration in spring volatile oil (0.14%). Monoterpenes were detected in *P. suaveolens* stem bark in small amount accounting for 2.2% of the total oil composition [35]. However, they weren’t detected in *P. sumatrana*, *P. stenopetalla*, *P. cauliflora*, and *P. rumphii* essential oils. The difference of the chemical composition among *Polyalthia* species could be due to the different stages of development, extraction procedures, and specific region where the plant was harvested [33].

Non-terpenic compounds represented 1.94–29.92% of the total volatile oil composition, with the highest concentration found in winter volatile oil sample (29.92%), followed by spring volatile oil (8.53%), then autumn volatile oil (5.22%), and the least concentration was detected in summer volatile oil (1.94%). 3-methyl heneicosane (0.21–3%) and 3-methyl tetracosane (0.51–2.99%) were the most abundant compounds of this class. Oxygenated sesquiterpenes represented 1.91–15.46% of the total volatile oil composition. Spring volatile oil exhibited the highest concentration (15.46%), meanwhile, winter volatile oil exhibited the least concentration (1.91%). The most abundant compound in this class was caryophyllene oxide (1.12–6.27%). Previous study on *P. michaelii* leaves essential oil revealed that the major compound was spathulenol (42.2%) [34].

Non-terpenic oxygenated compounds were detected in three seasons only, their concentrations were 8.02, 4.11, and 2% in summer, autumn, and spring volatile oils, respectively. Long chain aldehydes, esters and acids were detected, such as nonanal (C9, present in summer and spring volatile oils), oleic acid (present in autumn volatile oil), and palmitic acid, methyl ester (present in summer, autumn, and spring volatile oils).

Diterpenic hydrocarbons represented 0.59 to 5.32% of the total volatile oil composition. Four compounds were detected phytane, phytol, isoincensole, and verticilla-4(20),7,11-triene. Oxygenated monoterpenes were detected in summer (2.49%), autumn (0.73%), and spring (0.86%) volatile oils only. Borneol acetate (0.13–1.24%) was the most abundant member of this class. The last group contained a variety of classes such as *α*-ionone and (E)-*β*-damascenone (norisoprenoids, which are produced by oxidative cleavage of carotenoids and are responsible of the aroma of plants such as tobacco [36]). Sterols were also identified as *γ*-sitosterol and campesterol. Sterols were previously detected in family Annonaceae [37].

Previous study reported that *P. sessiliflora* stem oil contained eugenol as the predominant compound with a concentration of 42.7% of the total oil composition. It may be possible to postulate that every species has its own compositional pattern [34].

These findings corroborate the idea that seasonal variation can cause the variation of the composition of volatile oil and affect the oil yield [38]. Several factors can lead to the variation in the essential oil content and its composition, such as light, temperature, reproductive stage, season, and the growing conditions which is in accordance with results reported before [26, 39, 40]. From our findings, we have deduced that seasonal variation can cause alterations in both yield and chemical composition of the tested sample; the highest yield season was spring and the best season for volatile oil production.

### Acetylcholinesterase inhibitory activity

Medicinal plants and their essential oils have been a prominent source for various activities such as enzyme inhibition as a safer option than synthetic drugs [26, 41, 42]. *Polyalthia* essential oils have been reported to exert numerous biological activities including; cytotoxic [12, 43], antimicrobial [12, 44, 45], anti-inflammatory [43], and insecticidal activities [46].

Alzheimer’s disease (AD), a progressive neurodegenerative disorder, that affects the older and pre-elderly population. This neurological disorder issues from a shortage of acetylcholine (ACh) and is described by exacerbated brain tissue degeneration [47].

Acetylcholine, a neurotransmitter released by cholinergic neurons in synaptic gaps, is involved in memory and learning behaviors. Its decrease has been related to AD [48]. Inhibition of AChE, a cholinergic enzyme found at postsynaptic neuromuscular junctions that breaks down ACh into acetic acid and choline [49], can increase the level of acetylcholine in the brain alleviating the symptoms of the disease and improving cognitive function [50].

To the best of our knowledge, the AChE inhibitory activity of PSLO has never been reported before. However, some alkaloids isolated from *P. stenopetala and P. sumatrana* inhibited AChE with percentage inhibition values ranging from 40.2 to 80.6% [51, 52].

The percentage inhibition of 10 µg/mL and 100 µg/mL of PSLO were 16.15$\pm$2.17% and 51.44$\pm$2.8%, respectively **(**Table 2**)**. The inhibitory activity of different concentrations of standard (Donepezil) and essential oil is summarized in supplementary information (Fig. S2).

**Table 2:** Acetylcholinesterase inhibitory effect of *P. suberosa* leaf volatile oil

Sample	AChE inhibitory activity (%) *		IC_50_ **
	10 µg/mL	100 µg/mL	
PSLO	16.15$\pm$2.17%	51.44$\pm$2.8%	91.94 µg/mL
Donepezil	-	-	9.228 nM

PSLO: *P. suberosa* leaf volatile oil

All determinations are carried out in triplicate manner, and the values are represented as mean$\pm$SD

* Percentage inhibition calculated as previously mentioned in experimental section

** Concentration of sample used to inhibit 50% of acetylcholinesterase under the test conditions

According to Taqui et al. [53], plant extracts/fractions which have AChE inhibitory activity were classified into three categories based on their IC_50_ values: high potency, IC_50_ < 20 µg/mL; moderate potency, 20 < IC_50_ < 200 µg/mL; and low potency, 200 < IC_50_ < 1000 µg/mL. PSLO showed moderate potency against AChE with IC_50_ value of 91.94 µg/mL as shown in Table 2.

The anti-AChE activity of the tested oil may be attributed to the synergistic effect of its components, especially monoterpenes and sesquiterpenes. It has been reported that essential oils containing monoterpenes had the tendency to exhibit good AChE inhibitory effect [54]. D-Limonene was reported to exhibit potent AChE inhibition activity with IC_50_ value of 3.54 mM [55]. *α*-Pinene showed a strong AChE inhibitory activity with IC_50_ value of 0.022 mg/mL, meanwhile, *α*-terpineol showed a weak activity with IC_50_ value of 1.3 mg/mL [56]. Linalool and *γ*-terpinene exhibited weak inhibition of AChE [57]. In addition, several compounds were reported to exhibit anti-cholinesterase activity such as camphene [58], *p*-cymene [59], *β*-pinene, 2-carene, terpinolene, linalool [60], and sabinene [61]. A study showed that the effect of administration of myrcene, a monoterpene, alone or with donepezil significantly reversed the neurodegenerative effects of AlCl_3_ and D-galactose in mice, where myrcene enhanced the cholinergic activity and reduced neuroinflammation [62].

Liu et al. studied the interactions between the individual volatile components on AChE inhibition. The study showed that *α*-terpinolene displayed synergistic effect with sabinene, limonene, and *α*-pinene. Sabinene had synergistic effects with both limonene and 4-terpineol [63].

It was reported that essential oils containing mainly sesquiterpenes compared to the oils containing monoterpenes were more potent inhibitors of AChE activity, and the same was observed in the case of mixtures dominated by sesquiterpenes where they showed stronger inhibition than mixture dominated by monoterpenes [64]. For instance, a study reported that a combination of (E)-*β*-caryophyllene, *α*-pinene, and *α*-humulene (31:17:10) exhibited more potent inhibitory activity with IC_50_ value of 25 µg/mL compared to the *G. bicolor* leaf oil IC_50_ value of 85 µg/mL which contained the three major volatile components. Meanwhile, the stem oil of the *G. bicolor* containing *α*-pinene, *β*-pinene, and (E)-*β*-caryophyllene showed inhibitory activity with IC_50_ value of 92 µg/mL. A mixture of *α*-pinene, *β*-pinene, and (E)-*β*-caryophyllene (61:14:5) was more potent at inhibiting AChE activity than the stem oil [65].

*α*-Copaene, E-*β*-caryophyllene, *α*-humulene, and *α*-farnesene were reported to exhibit moderate AChE inhibitory activity [50]. Farnesene was reported to possess neuroprotective effect *via* significantly ameliorating the cytotoxicity of *β*-amyloid peptides and decreasing AChE activity [66].

Bonesi et al. reported that *trans*-caryophyllene inhibited AChE with a percentage of 32% at 0.06mM [61]. An in-vivo study showed that germacrene D significantly inhibited AChE in rat brain structures with percentage over 50% [67]. *α*-Copaene showed strong synergism combined with both (*E*)-*β*-caryophyllene and *α*-humulene [54].

Other compounds also were reported to possess promising AChE inhibitory activity such as viridiflorol and elemol that strongly inhibited AChE with IC_50_ values of 25 and 34 µg/mL, respectively [68]. Caryophyllene oxide showed strong AChE inhibitory activity [69]. Phytol improved cognitive functions in scopolamine-induced AD in rats by inhibiting AChE and butyrylcholinesterase enzyme [70]. Palmitic acid and squalene showed inhibition of AChE using TLC bio-autography assay [71].

The biological activity of the essential oil is due to the chemical complexity of the essential oil and the contribution of its individual constituents, since each constituent of this complex is included in the overall activity or may modulate the effects of the other constituents [72]. In this sense, we suggest further studies to isolate the oil components and test their AChE inhibitory activity as individuals.

These findings suggest a synergistic effect between different compounds identified in PSLO with higher contribution of certain components to this activity [63, 65], thus highlighting the use of the isolated oil as an adjuvant therapy in treatment of Alzheimer’s disease [73].

### *In-silico* molecular docking study

#### Molecular docking study

**Fig. 4:**
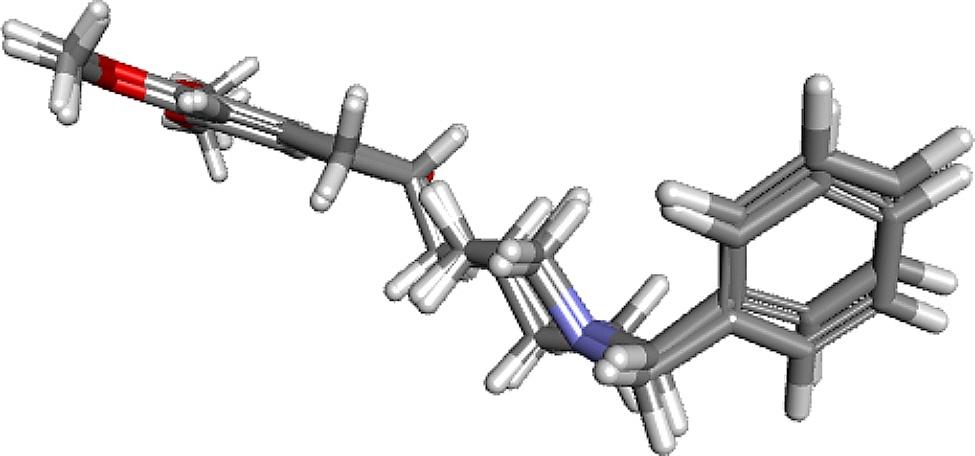
Validation of the docking protocol

PSLO exhibited notable AChE inhibitory activity; thus, an *in-silico* molecular docking study was carried out to corroborate the attained results. The human acetylcholinesterase crystal structure was obtained from the Protein Data Bank (http://www.rcsb.org/pdb/) complexed with donepezil (PDB ID 4EY7; 2.35 Å). Donepezil, the co-crystallized ligand, was utilized to identify the amino acid residues in the active site of acetylcholinesterase enzyme. The computed free binding energies ∆G (Kcal/mol) of major compounds found in PSLO were displayed in Table 3. Furthermore, the docking parameters were validated by re-docking the co-crystallized ligand into the active site of acetylcholinesterase. The calculated RMSD value between the co-crystallized ligand and the docked pose was 0.32 Å indicating the validity of the docking protocol (Fig. 4).

**Table 3:** Free binding energies (∆G) of the major identified compounds in *Polyalthia suberosa* leaf essential oil within the active sites of human acetylcholinesterase using molecular docking and expressed in Kcal/mol. Positive values indicate unfavorable interaction

	Compound name	C-Docker Energy ∆G (Kcal/mol)
1	Palmitic acid	-52.6742
2	**Donepezil (4EY7, co-crystallized inhibitor**	-28.9953
3	Phytol	-23.4315
4	*p*-Cymene	-21.6335
5	Caryophyllene oxide	-5.84702
6	*β*-Pinene	-0.384704
7	*α*-Copaene	1.72951
8	*α*-Pinene	1.93741
9	*α*-Terpineol	4.63067
10	Linalool	4.74992
11	Germacrene D	7.36147
12	Myrcene	9.10231
13	*E*-*β*-Caryophyllene	9.58014
14	Viridiflorol	12.5957
15	2-Carene	13.0745
16	Elemol	14.3889
17	D-limonene	16.8749
18	*β*-Elemene	18.5104
19	Bicyclogermacrene	21.889
20	*γ*-Terpinene	22.0405
21	*α*-Farnesene	27.5747
22	Terpinolene	32.4463
23	Camphene	43.5986
24	*α*-Humulene	49.2467
25	*γ*-Sitosterol	68.1062
26	Squalene	90.07
27	24-Noroleana-3,12-diene	161.718

Palmitic acid, phytol, *p*-cymene, and caryophyllene oxide showed the best affinity and fitting (Supplementary information Fig. S3), displaying free binding energy values of -52.6742, -23.4315, -21.6335, and − 5.84702 Kcal/mol, respectively, with palmitic acid exceeding the value of the standard drug donepezil which exhibited ∆G equals to -28.9953 Kcal/mol.

AChE active site, located at the centre bottom of the enzyme molecule, is a 20 Å deep gorge. The active site consists of several subsites. These important sites and residues are catalytic triad (Ser203, His447, Glu334), anionic subsite (Trp86, Tyr133, Glu202, Gly448, Ile451), oxyanion hole (Gly121, Gly122, Ala204), acyl binding pocket (Trp236, Phe295, Phe297, Phe338) and peripheral anionic subsite (Asp74, Tyr124, Ser125, Trp286, Tyr337, Tyr341) [74].

The high fitting scores of these compounds within the active site of AChE can be clarified by their agreeable binding through the formation of various bonds. Palmitic acid formed one conventional hydrogen bond with Gly120. Meanwhile, phytol formed two conventional hydrogen bonds with Tyr133 and Glu202 and six π-alkyl bonds with Trp86, Tyr337, Phe338, Tyr341, and Tyr72. *p*-Cymene formed four π-alkyl bonds with Phe338, Tyr337, and Tyr341 in addition to one π-lone pair bond with Tyr124. Caryophyllene oxide formed one π-δ bond with Trp86 and six π-alkyl bonds with Trp86, His447, Phe297, and Phe338. Donepezil, the co-crystallized inhibitor, was used as the reference acetylcholinesterase inhibitor drug and formed two hydrogen-water bonds with HOH253, one conventional hydrogen bond with Phe295, two π-alkyl bonds with Tyr337 and Tyr341, two π-δ bonds with Tyr341 and Phe338, three π-π bonds with Trp86, Trp286, and Tyr34, and three hydrogen-carbon bonds with Ser293 and Tyr341. Noteworthy, all these compounds exhibited Van der Waals forces with the amino acid residues present in the active binding site of AChE.

### ADMET

The aim of ADMET prediction, an important step in pharmaceutical R&D development, is to explore the drug-like properties of the identified compounds in PSLO. As shown in supplementary information (Table S1), most of the identified compounds in PSLO displayed very high and high penetration through blood brain barrier (BBB) which is important for the inhibition of AChE in the brain except for phytol, squalene, 24-noroleana-3,12-diene, and *γ*-sitosterol. Most compounds showed good and moderate intestinal absorption except phytol, squalene, 24-noroleana-3,12-diene, and *γ*-sitosterol. The compounds exhibited a range of water solubility varying from very poor solubility to good solubility.

Few compounds such as 24-noroleana-3,12-diene, *α*-copaene, camphene, caryophyllene oxide, *E*-*β*-caryophyllene, and *γ*-sitosterol exhibited certain hepatotoxicity. Fortunately, all the compounds were non-inhibitors for CYP2D6 except donepezil and germacrene D, thus, no drug-drug or drug-herb interactions would be encountered. However, most of the compounds exhibited plasma protein binding (PPB) leading to a decrease in free plasma fraction thus a decrease in the volume of distribution and decreasing the concentration of the drug at the site of action.

**Fig. 5:**
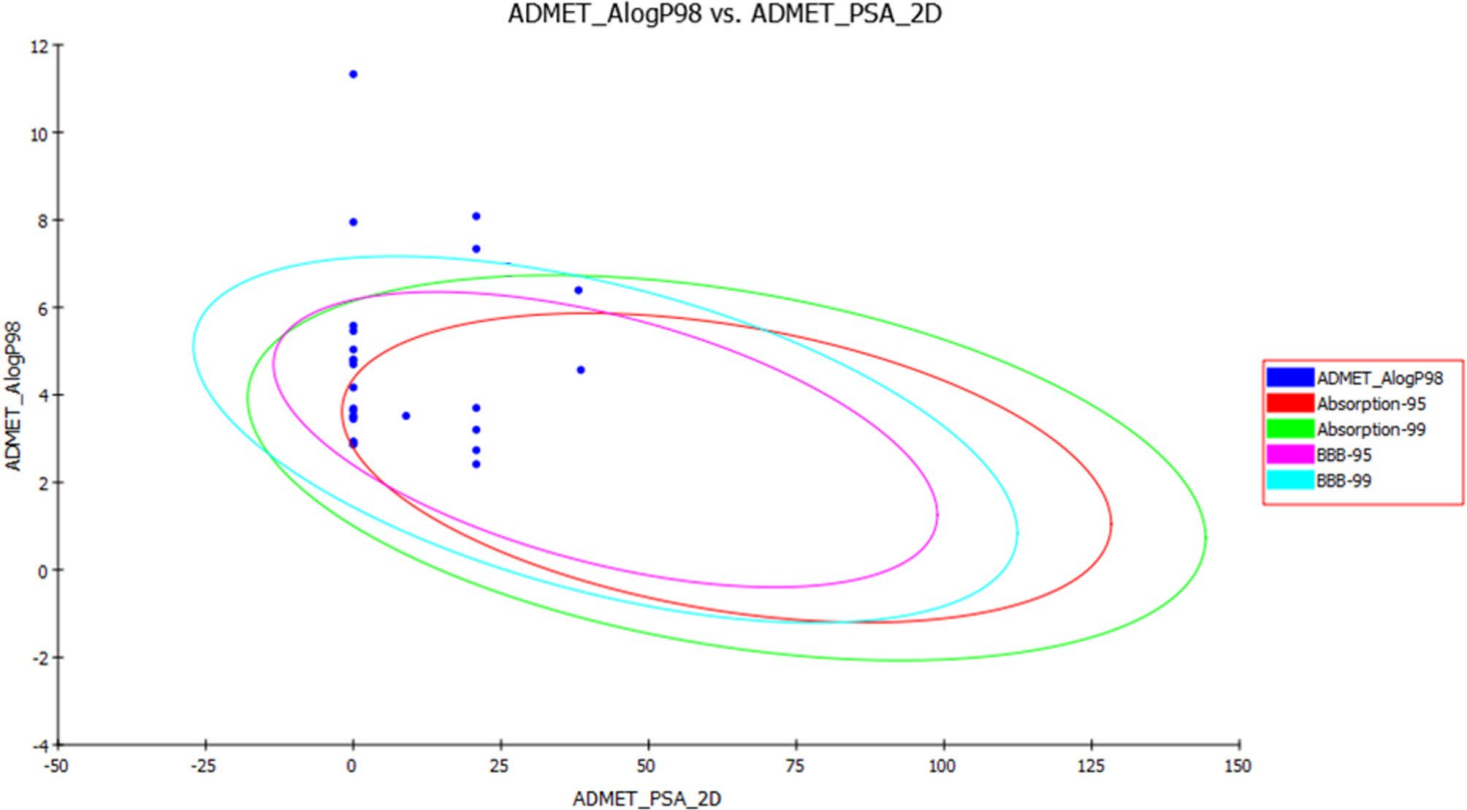
ADMET Plot for bioactive metabolites identified in *P. suberosa* leaf essential oil displaying 95% and 99% confidence limit ellipses corresponding to blood-brain barrier (BBB) and human intestinal absorption models

Those results are shown in ADMET plot **(**Fig. 5**)**, where all compounds showed excellent intestinal absorption and blood-brain barrier penetration, as evidenced by their allocation in the 99% absorption ellipse. Concomitantly, phytol, squalene, 24-noroleana-3,12-diene, and *γ*-sitosterol that showed poor intestinal absorption and an undefined BBB penetration were positioned outside the 99% absorption ellipse.

## Conclusion

Chemical profiling of the *P. suberosa* leaf volatile oils obtained in four different seasons unveiled that the composition and yield of the oil were varied according to seasonal changes. PSLO showed moderate acetylcholinesterase inhibition, this may be due to synergism between certain components of the oil. *In-silico* molecular docking unveiled that palmitic acid, phytol, *p*-cymene, and caryophyllene oxide demonstrated the best fitting scores within the active sites of human acetylcholinesterase enzyme. To the best of our knowledge, this is the first study to highlight the promising use of *P. suberosa* leaf essential oil as an adjuvant therapy in the management of Alzheimer’s disease. Further *in-vivo* neuroprotective investigations and validation of the isolated essential oil are recommended.

### Electronic supplementary material

Below is the link to the electronic supplementary material.

Supplementary Material 1

## Data Availability

Data are available upon request from the firstauthor.

## Abbreviations

ACh: Acetylcholine
AChE: Acetylcholinesterase enzyme
AD: Alzheimer’s disease
ADMET: Absorption, distribution, metabolism, excretion, and toxicity
BBB: Blood brain barrier
DMSO: Dimethyl sulfoxide
DTNB: 3,3′-dithiodipropionic acid di-(N-hydroxysuccinimide ester)
GC/MS: Gas Chromatography/Mass Spectrometry
PPB: Plasma protein binding
PSLO: *P. suberosa* leaf essential oil
RMSD: Root mean square deviation
TLC: Thin layer chromatography
