# Supplementary material for: The impact of seasonal variation on the composition of the volatile oil of Polyalthia suberosa (Roxb.) Thwaites leaves and evaluation of its acetylcholinesterase inhibitory activity
Source: BMC Complement Med Ther. 2024 Apr 12;24:159. doi: 10.1186/s12906-024-04443-z (PMC11015646; doi:10.1186/s12906-024-04443-z)
Supplement: Supplementary file 1 — Supplementary Material 1 [file 12906_2024_4443_MOESM1_ESM.docx]

Supplementary Material 1

Content

| No. | Title |
| --- | --- |
| Fig. S1 | GC-MS chromatograms of *P. suberosa* volatile constituents in four different seasons; (A) summer season, (B) autumn season, (C) winter season, and (D) spring season. |
| Fig. S2 | Dose-dependent AChE percentage inhibition of (A) donepezil and (B) *P. suberosa* leaf essential oil |
| Fig. S3 | 2D and 3D binding modes of palmitic acid (A), phytol (B), p-cymene(C), caryophyllene oxide (D) and donepezil (E) within the active sites of human acetylcholinesterase employing C-docker protocol. |
| Table S1 | Absorption, distribution, metabolism, excretion, and toxicity (ADMET) properties of major metabolites identified in *Polyalthia suberosa* leaf essential oil |


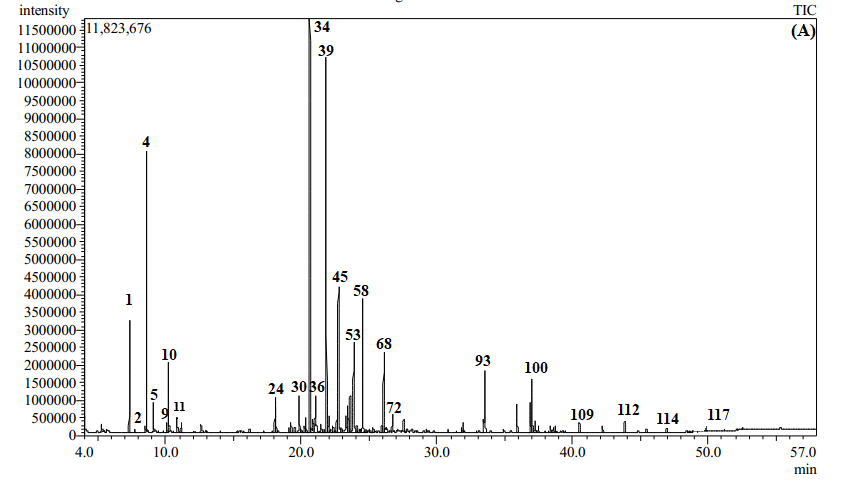


**
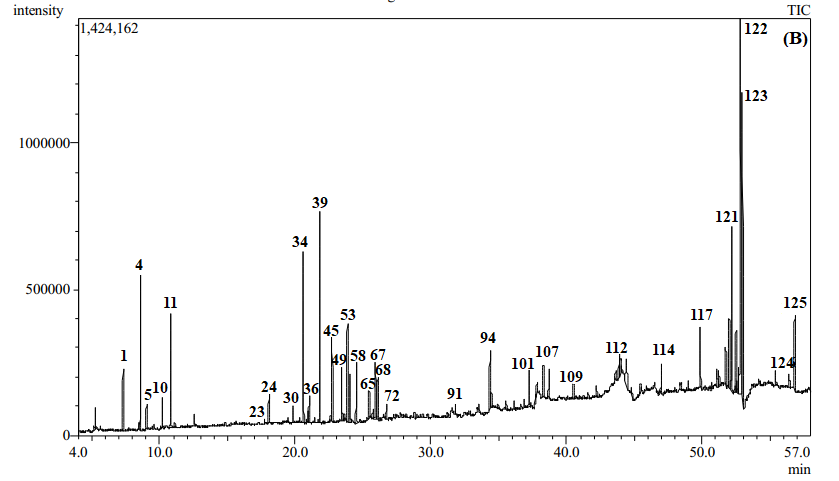
**


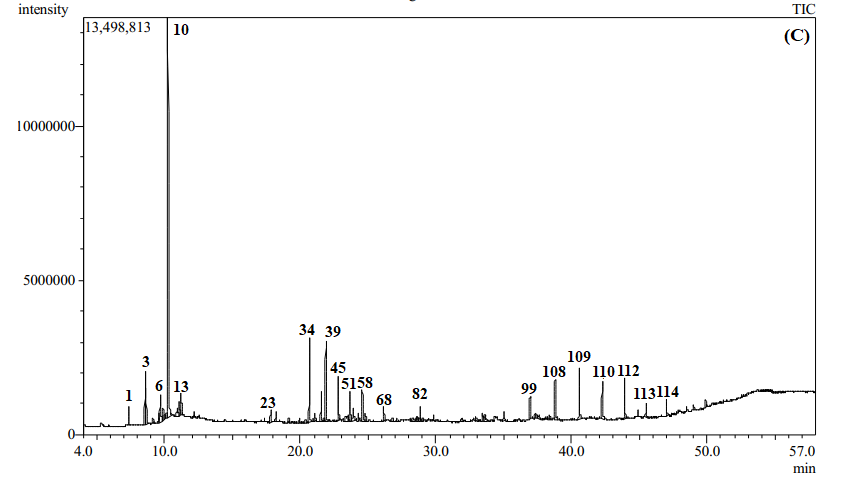


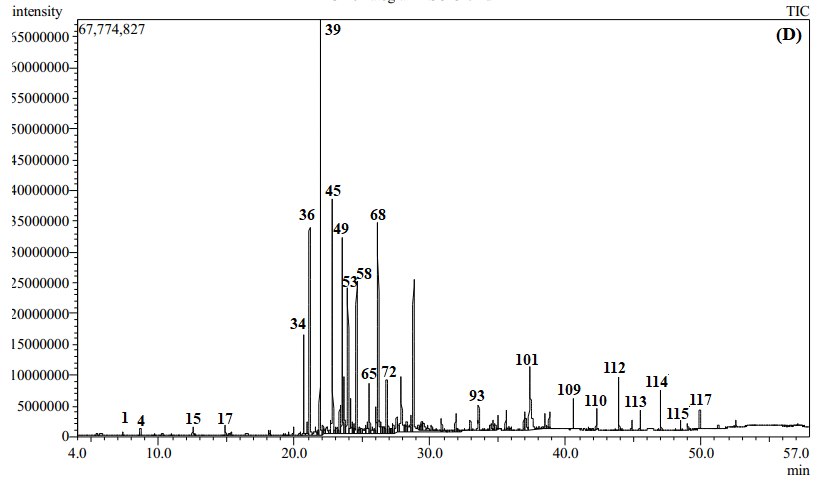


**Figure S1.** GC-MS chromatograms of *P. suberosa* volatile constituents in four different seasons; (A) summer season, (B) autumn season, (C) winter season, and (D) spring season.


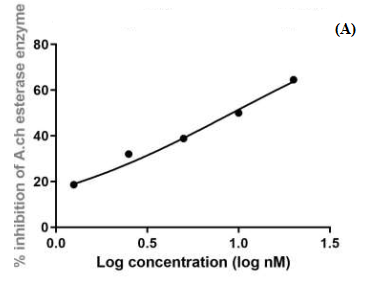


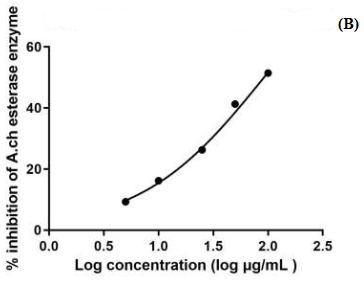


**Figure S2.** Dose-dependent AChE percentage inhibition of (A) donepezil and (B) *P. suberosa* leaf essential oil.

| 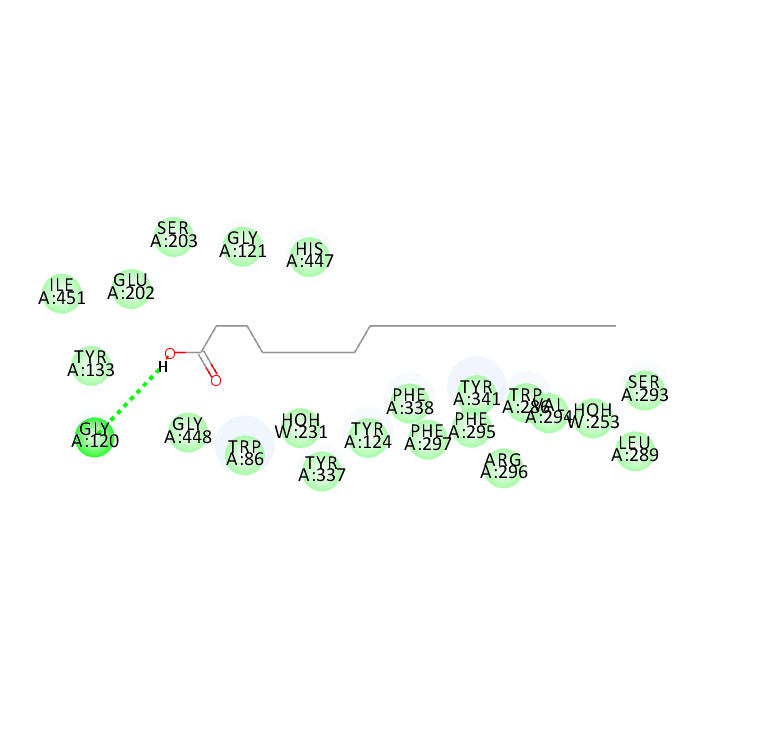  **(A)**  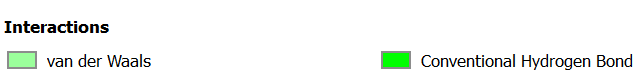 | 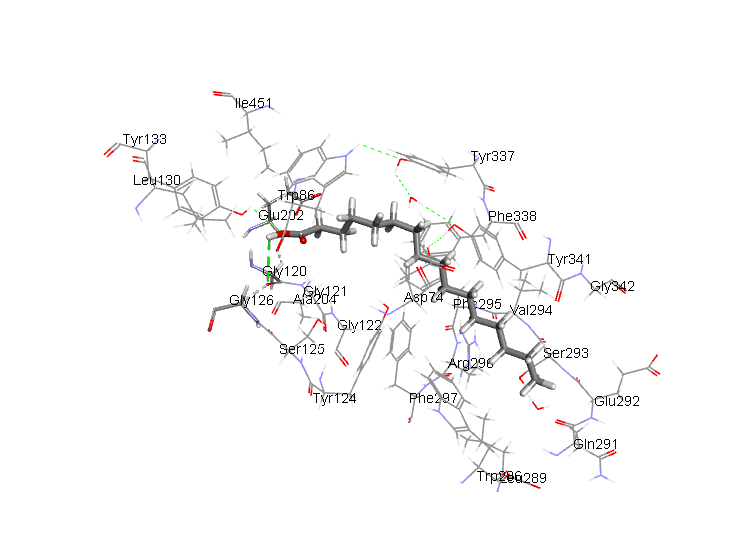 |
| --- | --- |
| 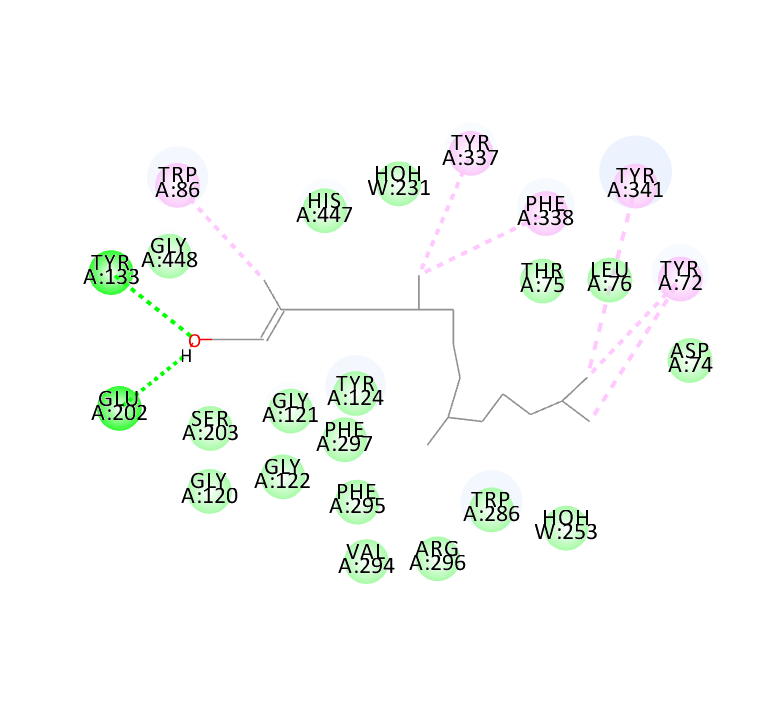  **(B)**  **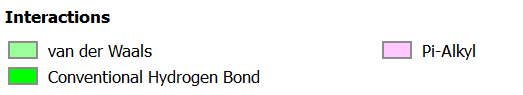** | 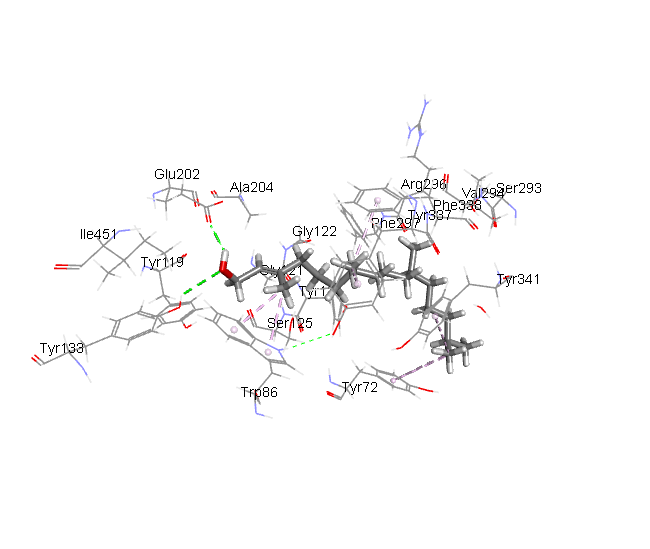 |
| 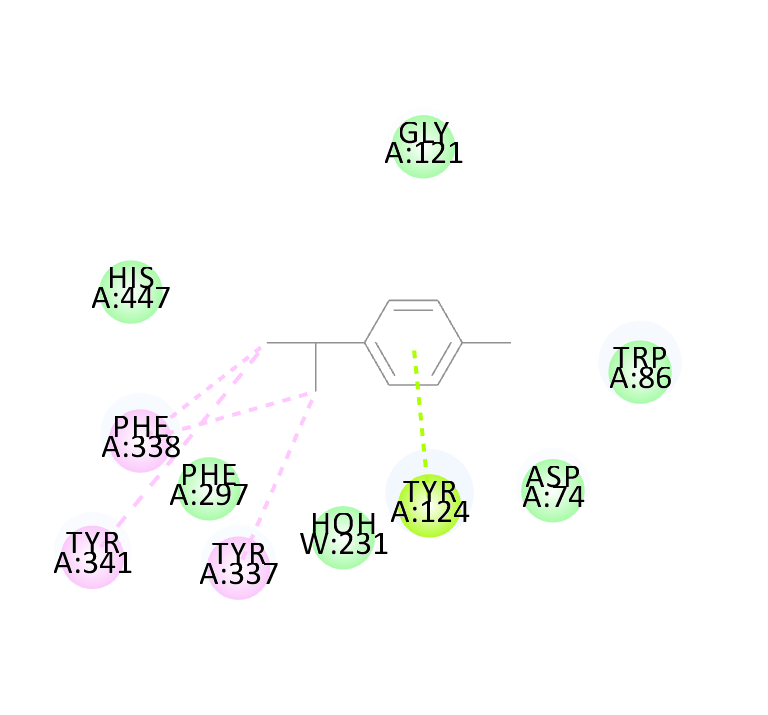  **(C)**  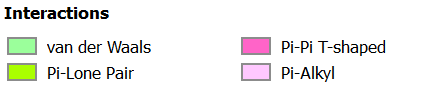 | 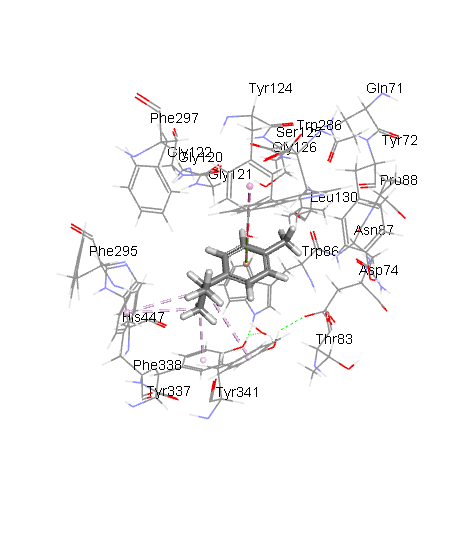 |
| 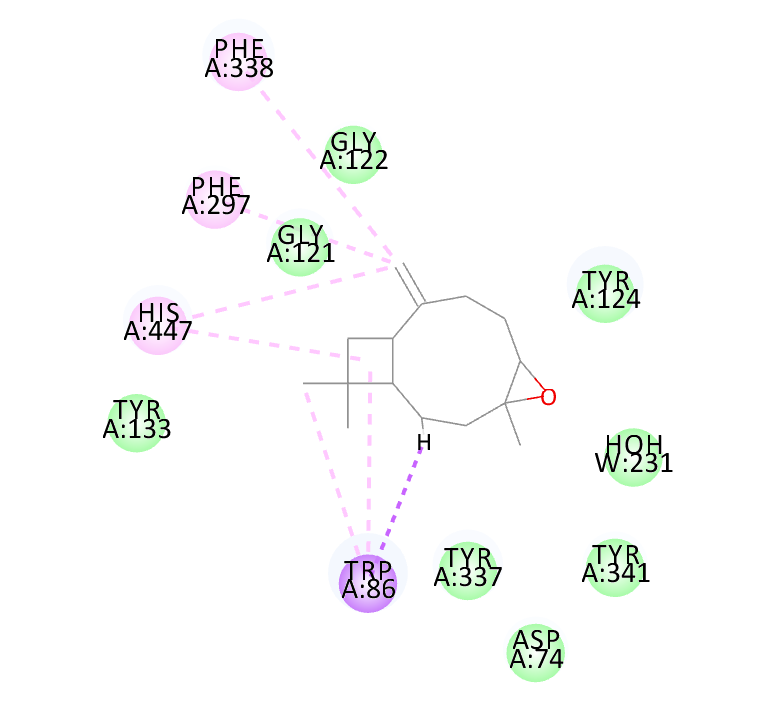  **(D)**  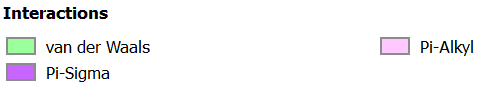 | 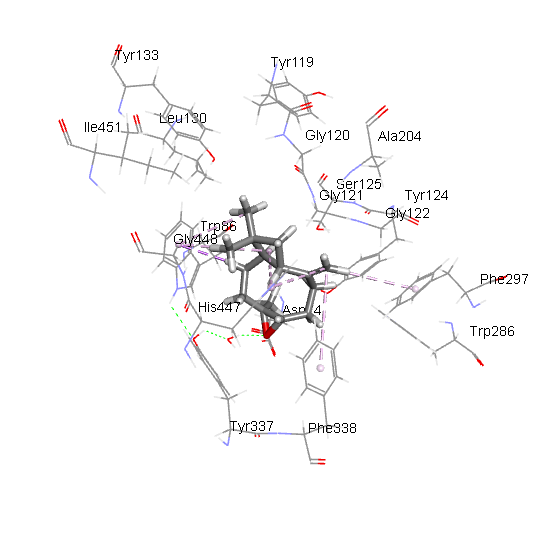 |
| 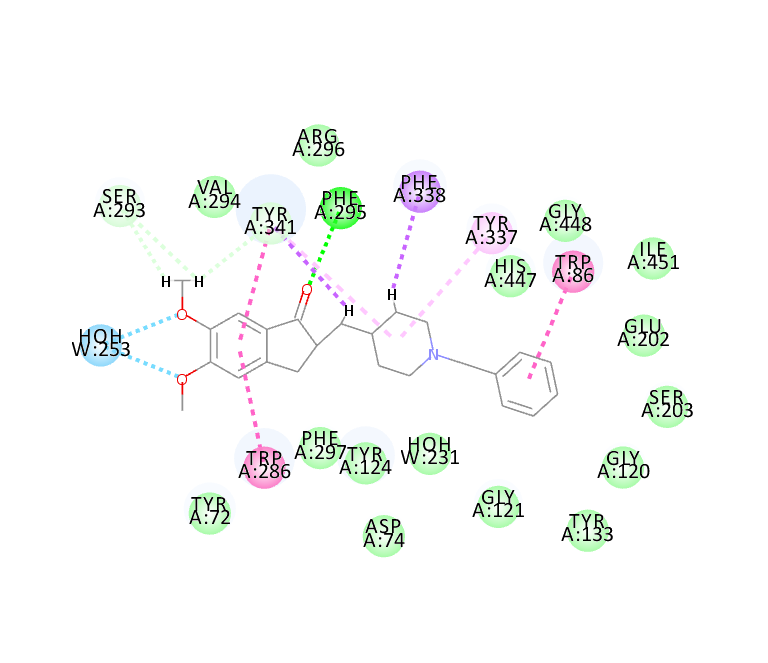  **(E)**  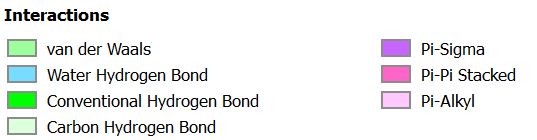 | 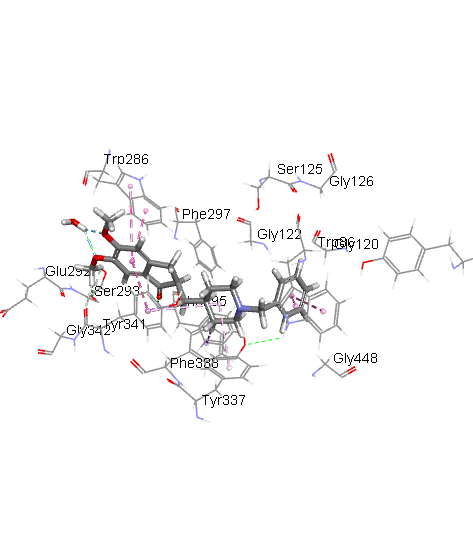 |

**Figure S3.** 2D and 3D binding modes of palmitic acid (A), phytol (B), p-cymene(C), caryophyllene oxide (D) and donepezil (E) within the active sites of human acetylcholinesterase employing C-docker protocol

**Table S1.** Absorption, distribution, metabolism, excretion, and toxicity (ADMET) properties of major metabolites identified in *Polyalthia suberosa* leaf essential oil

| **Compound name** | **BBB level** | **Absorption level** | **Solubility level** | **Hepato-toxicity** | **CYP2D6** | **PPB level** | **AlogP98** | **PSA 2D** |
| --- | --- | --- | --- | --- | --- | --- | --- | --- |
| Donepezil | 1 | 0 | 2 | 0 | 1 | 1 | 4.569 | 38.513 |
| Linalool **(15)** | 1 | 0 | 3 | 0 | 0 | 0 | 2.735 | 20.815 |
| Myrcene **(5)** | 0 | 0 | 3 | 0 | 0 | 0 | 3.688 | 0 |
| **Palmitic acid (94)** | 0 | 1 | 2 | 0 | 0 | 2 | 6.392 | 38.116 |
| ***p*-Cymene (9)** | 0 | 0 | 3 | 0 | 0 | 2 | 3.51 | 0 |
| **Phytol (101)** | 4 | 3 | 2 | 0 | 0 | 2 | 7.337 | 20.815 |
| Squalene **(116)** | 4 | 3 | 0 | 0 | 0 | 2 | 11.331 | 0 |
| Terpinolene **(14)** | 0 | 0 | 2 | 0 | 0 | 0 | 3.643 | 0 |
| Viridiflorol **(71)** | 1 | 0 | 2 | 0 | 0 | 0 | 3.202 | 20.815 |
| 2-Carene **(7)** | 0 | 0 | 3 | 0 | 0 | 0 | 2.872 | 0 |
| 24-Noroleana-3,12-diene **(116)** | 4 | 3 | 0 | 1 | 0 | 2 | 7.949 | 0 |
| *α*-Copaene **(34)** | 0 | 0 | 2 | 1 | 0 | 1 | 4.168 | 0 |
| *α*-Farnesene **(54)** | 0 | 1 | 2 | 0 | 0 | 2 | 5.46 | 0 |
| *α*-Humulene **(45)** | 0 | 1 | 2 | 0 | 0 | 2 | 5.035 | 0 |
| *α*-Terpineol **(18)** | 1 | 0 | 3 | 0 | 0 | 0 | 2.415 | 20.815 |
| *α*-Pinene **(1)** | 0 | 0 | 3 | 0 | 0 | 0 | 2.872 | 0 |
| *β*-Elemene **(36)** | 0 | 1 | 2 | 0 | 0 | 1 | 4.788 | 0 |
| ***β-*Pinene (4)** | 0 | 0 | 3 | 0 | 0 | 0 | 2.926 | 0 |
| Bicyclogermacrene **(53)** | 0 | 1 | 2 | 0 | 0 | 1 | 4.699 | 0 |
| Camphene **(2)** | 0 | 0 | 3 | 1 | 0 | 0 | 2.926 | 0 |
| **Caryophyllene oxide (68)** | 0 | 0 | 2 | 1 | 0 | 0 | 3.519 | 8.93 |
| D-limonene **(10)** | 0 | 0 | 3 | 0 | 0 | 0 | 3.502 | 0 |
| *E*-*β*-caryophyllene **(39)** | 0 | 1 | 2 | 1 | 0 | 1 | 4.807 | 0 |
| Elemol **(63)** | 1 | 0 | 3 | 0 | 0 | 0 | 3.7 | 20.815 |
| *γ*-Sitosterol **(124)** | 4 | 3 | 0 | 1 | 0 | 2 | 8.084 | 20.815 |
| *γ-*Terpinene **(13)** | 0 | 0 | 3 | 0 | 0 | 2 | 3.448 | 0 |
| Germacrene D **(49)** | 0 | 1 | 1 | 0 | 1 | 2 | 5.58 | 0 |

0, 1, 2, 3, and 4 denote very high, high, medium, low, and undefined penetration *via* BBB respectively. 0, 1, 2, and 3 signify good, moderate, poor, and very poor intestinal absorption, respectively. Aqueous solubility: 0, 1, 2, 3, 4, and 5 show extremely low, very low but possible, low, good, optimal, and too soluble, respectively. Hepatotoxicity: 0, Non-toxic; 1, Toxic. CYP2D6, cytochrome P450-14DM inhibition: 0, non-inhibitor; 1, inhibitor. PBB, plasma protein binding: 0, less than 90%; 1, more than 90%. AlogP98, atom-type partition coefficient (ALogP98). PSA 2D, 2D polar surface area in Å^2^
